# Present and Future of Central Disorders of Hypersomnolence

**DOI:** 10.1111/jsr.70118

**Published:** 2025-06-18

**Authors:** Francesco Biscarini, Lucie Barateau, Fabio Pizza, Giuseppe Plazzi, Yves Dauvilliers

**Affiliations:** ^1^ Department of Biomedical and Neuromotor Sciences (DIBINEM) University of Bologna Bologna Italy; ^2^ IRCCS Istituto delle Scienze Neurologiche di Bologna Bologna Italy; ^3^ Sleep‐Wake Disorders Unit, Department of Neurology, Gui‐de‐Chauliac Hospital CHU Montpellier Montpellier France; ^4^ National Reference Centre for Orphan Diseases, Narcolepsy, Idiopathic Hypersomnia, and Kleine‐Levin Syndrome Montpellier France; ^5^ Institute for Neurosciences of Montpellier University of Montpellier, INSERM Montpellier France; ^6^ Department of Biomedical, Metabolic and Neural Sciences University of Modena and Reggio‐Emilia Modena Italy

**Keywords:** excessive daytime sleepiness, hypersomnia, Kleine‐Levin syndrome, narcolepsy, orexin/hypocretin, perspectives

## Abstract

Central disorders of hypersomnolence (CDH) are rare neurological conditions lumped by excessive daytime sleepiness (EDS) as primary complaint mostly arising at young age, including narcolepsy type 1 (NT1), narcolepsy type 2 (NT2), idiopathic hypersomnia (IH), and Kleine‐Levin syndrome (KLS). Advances in clinical and translational research have deepened our understanding of NT1, particularly the loss of hypothalamic hypocretin/orexin‐producing neurons, establishing hypocretin deficiency as a reliable disease specific biomarker, although the exact mechanisms of neuronal loss remain unknown. Conversely, the pathophysiological mechanisms underlying NT2, IH, and KLS are still poorly understood, as well as their natural course. Standard diagnostic evaluation primarily relies on clinical symptoms, polysomnography and the multiple sleep latency test, with alternative neurophysiological markers (long term polysomnographic recordings), quantitative signal analysis, and wearables technologies being explored as potential innovative tools. Management remains symptomatic, combining pharmacological treatments such as stimulants, sodium oxybate, and emerging hypocretin/orexin receptor agonists, with nonpharmacological strategies tailored to improve patient quality of life. Notably, new therapies targeting orexin signalling offer promising avenues for transforming treatment approaches, particularly in NT1. Looking ahead, advancing precision medicine approaches and addressing unmet needs in CDH are essential to improve patient outcomes. This review summarises current knowledge and highlights future research directions in CDH pathophysiology, diagnostic approaches and pharmacological management.

## 1 Introduction

Central disorders of hypersomnolence (CDH) are a group of neurological conditions mainly characterised by excessive daytime sleepiness (EDS) not explained by a sleep or circadian sleep–wake rhythm disorder (American Academy of Sleep Medicine 2023). According to the current Third Edition of the International Classification of Sleep Disorders—Text Revision (ICSD‐3‐TR), the conditions accounted among CDH are narcolepsy type 1 (NT1), narcolepsy type 2 (NT2), idiopathic hypersomnia (IH), Klein‐Levin Syndrome (KLS), insufficient sleep syndrome (ISS), hypersomnia due to a medical disorder, hypersomnia due to a medication or substance, and hypersomnia associated with a psychiatric disorder (American Academy of Sleep Medicine 2023). In particular, NT1, NT2, IH, and KLS are rare diseases considered central hypersomnolence disorders, in which EDS is an intrinsic cerebral dysfunction not related to any other factor, condition, or behaviour.

NT1, formerly named narcolepsy with cataplexy (American Academy of Sleep Medicine 2005), is characterised by (i) EDS and one of the following criteria: (ii) cataplexy and a mean sleep latency (mSL) of ≤ 8 min with ≥ 2 sleep‐onset rapid eye movement (REM) periods (SOREMP, i.e., REM sleep occurring within 15 min from sleep onset) on the multiple sleep latency test (MSLT), (iii) cataplexy and a SOREMP on a nocturnal polysomnography (PSG), or (iv) cerebrospinal fluid (CSF) hypocretin‐1 (CSF‐HCRT1) levels ≤ 110 pg/mL measured with radio‐immuno‐assay (RIA) according to the standard reference of the Stanford values (or < 1/3 of mean values obtained in normal subjects with the same standardised assay) (American Academy of Sleep Medicine 2023).

NT2, formerly named narcolepsy without cataplexy (American Academy of Sleep Medicine 2005), is characterised by (i) EDS present for at least 3 months, (ii) mSL ≤ 8 min and ≥ 2 SOREMPs on the MSLT, with a SOREMP on the preceding nocturnal PSG that may replace one of the SOREMPs on the MSLT, (iii) absence of cataplexy, (iv) CSF‐HCRT1 > 110 pg/mL (or > 1/3 of normal values), if measured, and (v) hypersomnolence and/or MSLT findings are not better explained by other causes (American Academy of Sleep Medicine 2023).

IH is defined by (i) EDS lasting for at least 3 months, (ii) absence of cataplexy, (iii) PSG and MSLT not consistent with diagnosis of NT1 or NT2, either (iv) mSL of ≤ 8 min on the MSLT with a maximum of 1 SOREMP, or (v) total 24‐h sleep time is ≥ 660 min recorded on 24‐h PSG monitoring, or estimated by wrist actigraphy associated with a sleep log (averaged over at least 7 days with unrestricted sleep), (vi) ISS is ruled out, and (vii) hypersomnolence and/or MSLT findings are not better explained by other causes (American Academy of Sleep Medicine 2023).

KLS, also known as recurrent or periodic hypersomnia, is diagnosed when (i) the patient experiences at least two recurrent episodes of EDS or excessive sleep duration, each lasting between 2 days and 5 weeks, (ii) episodes recur at least once every 18 months, (iii) patient has normal or near normal sleep and wakefulness, cognition, behaviour, and mood between episodes, at least during the first years of the syndrome, (iv) during the episodes, the patient also experiences cognitive dysfunction, derealisation, major apathy, or disinhibited behaviour, and (v) EDS and cognitive and psychiatric symptoms are not explained by other conditions or use of drugs.

**FIGURE 1:**
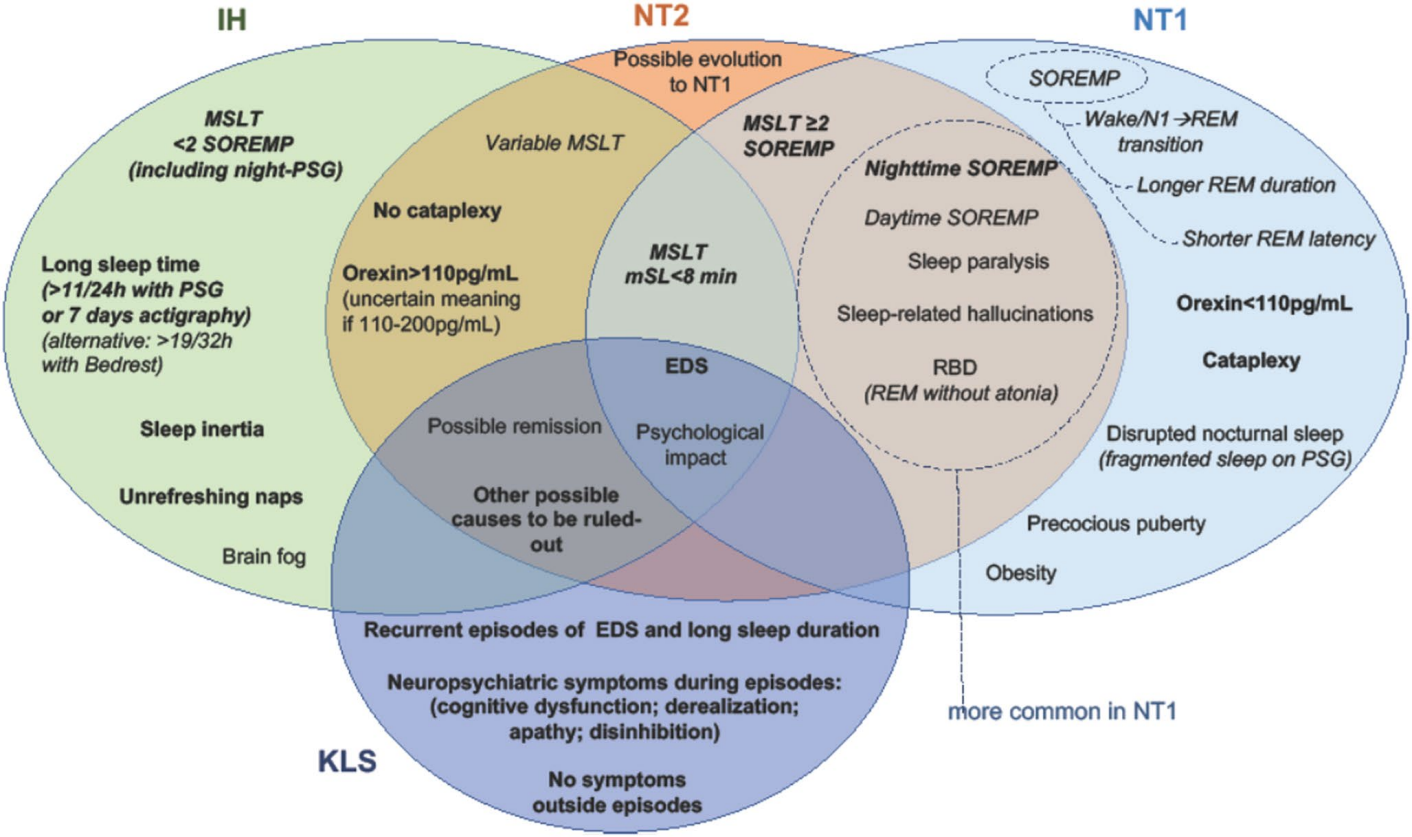
Shared and distinguishing features of central disorder of hypersomnolence (re‐adapted from Dauvilliers, Bogan, et al. (2022)). In bold = features part of current diagnostic criteria or supportive criteria in the International Classification of Sleep Disorders 3rd edition—Text Revision; in italics = neurophysiological features. EDS = excessive daytime sleepiness, mSL = mean sleep latency, MSLT = multiple sleep latency test, N1 = stage 1, NT1 = narcolepsy type 1, NT2 = narcolepsy type 2, PSG = polysomnography, RBD = rapid eye movement sleep behaviour disorder, REM = rapid eye movement sleep, SOREMP = sleep onset rapid eye movement period.

Over the years, clinical and pre‐clinical research on CDH has widely expanded, with consequent improvement in the understanding and management of these rare conditions. Scientific progress especially involved NT1. The acquired loss of the neuropeptide hypocretin/orexin due to immune‐mediated damage to the producing neurons in the hypothalamus was identified as the core pathological mechanism (Nishino et al. 2000; Thannickal et al. 2000; Peyron et al. 2000; Liblau et al. 2024; Latorre et al. 2018). The deficiency of HCRT1 in the CSF is a reliable and unique disease specific biomarker, and cataplexy a pathognomonic symptom of NT1 that helped to define the boundaries of this condition and go deeper into the clinical and neurophysiological phenotyping. Treatment has also improved towards a more targeted approach, up to the recent ongoing development of hypocretin‐receptor‐2 agonists (Dauvilliers, Mignot, et al. 2023; Evans et al. 2022). Research on other CDH is expanding, and the management of patients is improving, although the access to pharmacological treatments is still problematic in several countries, mainly for IH, and KLS. Furthermore, the pathological bases and course of NT2, IH, and KLS are still poorly understood (Figure 1).

The present review aims to present the current knowledge of CDH pathophysiology, diagnostic approaches and pharmacological, focusing on NT1, NT2, IH, and KLS. Based on the up‐to‐date evidence on CDH, the review suggests the possible future directions for research agenda.

## 2 Physiopathology

### 2.1 Narcolepsy Type 1

In 2000, a significant breakthrough shed light on the pathophysiology of NT1 when researchers discovered that this rare disorder is linked to a loss of a specific population of hypothalamic neurons responsible for producing orexin/hypocretin (Thannickal et al. 2000; Peyron et al. 2000). Post‐mortem analyses of the brains of individuals with narcolepsy and cataplexy demonstrated a selective loss of orexinergic neurons in the lateral hypothalamus. Orexin‐A and ‐B (also called hypocretin‐1 and ‐2) are neuropeptides essential for maintaining wakefulness (De Lecea et al. 1998; Sakurai et al. 1998). This neuronal population is relatively small (approximately 70,000–80,000 cells), but it has extensive projections throughout the brain. Consequently, orexin plays a fundamental role not only in sleep–wake regulation but also in neuroendocrine control, autonomic functions, reward processing, emotional regulation, and metabolism (Peyron et al. 1998). Across multiple species, a disruption in orexin signalling has been shown to induce a narcoleptic phenotype (Chemelli et al. 1999). The first animal model identified was a narcoleptic dog carrying a mutation in the gene encoding the orexin‐2 receptor (Lin et al. 1999). This discovery was instrumental in uncovering the aetiology of narcolepsy in humans (Thannickal et al. 2000; Peyron et al. 2000). While the absence of orexin is likely the primary driver of NT1, alterations in other neurotransmitter systems may also contribute to the symptomatology of the disorder. However, studies comparing CSF levels of monoamines, their metabolites, and trace amines between narcoleptic patients and individuals without EDS have not identified significant differences (Barateau, Jaussent, et al. 2021). Interestingly, NT1 patients exhibit an increased number of histaminergic neurons (Valko et al. 2013), though findings from narcoleptic mouse models have been inconsistent regarding this phenomenon (Shan et al. 2015). A recent post‐mortem analysis also revealed a selective reduction of corticotropin‐releasing hormone‐expressing neurons in the paraventricular nucleus of NT1 patients (Shan et al. 2022), alongside evidence of heightened histaminergic neuron activity (Shan et al. 2023).

The precise mechanism underlying the loss of orexin neurons remains unclear. However, the scientific community widely acknowledges that NT1 is likely an immune‐mediated disorder (Kornum et al. 2017; Barateau et al. 2017). This hypothesis is strongly supported by its remarkably close association with the HLA class II allele HLA‐DQB1*06:02 (present in over 98% of NT1 cases), as well as other genetic findings, including polymorphisms in T cell receptor and other key genes involved in immune function (Ollila et al. 2023). Additionally, the increased incidence of NT1 following Pandemrix vaccination during the 2009 H1N1 influenza pandemic further reinforces this hypothesis (Liblau et al. 2024). Current theories suggest that in genetically predisposed individuals, environmental factors such as viral or bacterial infections may trigger an immune response, activating CD4+ and CD8+ T cells. These cells exhibit a proinflammatory profile with elevated levels of cytokines, ultimately leading to the destruction of orexin‐producing neurons. The identification of self or foreign antigens targeted by the pathogenic T cell response in NT1 remains an active area of research. While increased T cell reactivity against orexin has been observed in NT1, conclusive evidence directly linking T cells to neuronal destruction is still lacking. However, recent studies in sera and CSF continue to provide valuable insights into the roles of autoreactive CD4+ and CD8+ T cells in NT1 (Latorre et al. 2018; Pedersen et al. 2019; Luo et al. 2018; Hartmann et al. 2016; Beltrán et al. 2019; Huth et al. 2024). On the other hand, a recent study found no evidence of in vivo increased microglia density in NT1 compared with controls, even close to disease onset (Barateau, Krache, et al. 2024a). The novel finding of increased circulating cell‐free mitochondrial DNA in the CSF of people with NT1, negatively correlated with CSF‐HCRT1, suggests the presence of mitochondrial dysfunction as part of the pathogenic process (Moresco et al. 2025). In the end, the mystery remains.

### 2.2 Narcolepsy Type 2

Initially regarded as a phenotypic variant of narcolepsy with cataplexy (and previously named ‘narcolepsy without cataplexy’), NT2 is now recognised as a distinct clinical entity. In contrast to NT1, its pathophysiological mechanisms are not understood, likely due to the disorder's heterogeneous and multifaceted nature (Bassetti et al. 2019). The clinical trajectory of NT2 may vary among patients. Some individuals eventually develop cataplexy and are reclassified as NT1, particularly if low CSF orexin levels are detected (Pizza et al. 2014). Others maintain a stable chronic condition, while some experience spontaneous improvement. Sometimes, the phenotype may evolve toward idiopathic hypersomnia (Lopez et al. 2017; Trotti 2020). The association with HLA DQB1*06:02 is weaker than in NT1, and per definition CSF orexin‐A levels are higher than 110 pg/mL. Patients with narcolepsy who exhibit intermediate orexin levels are rare and present with a heterogeneous phenotype that does not clearly fit into either the NT1 or NT2 classification (Postiglione et al. 2022). The presence of HLA‐DQB1*06:02 and lower CSF orexin levels has been linked to typical narcolepsy features, highlighting the need for further research to differentiate incomplete forms of the disorder from probable secondary narcolepsy. Some researchers have proposed that NT2 patients may experience a partial degeneration of orexin neurons, suggesting a pathophysiological continuum between NT1 and NT2 (Gerashchenko et al. 2003; Thannickal et al. 2009). While most individuals with narcolepsy without cataplexy exhibit normal CSF orexin concentrations (Dauvilliers, Baumann, et al. 2003), a subset of NT2 patients has been reported to have orexin neuron loss below 50% of normal levels, a pattern also observed in rodent studies (Thannickal et al. 2009; Black et al. 2018). Notably, animal models with partial orexin depletion exhibit excessive sleepiness but do not develop cataplexy (Gerashchenko et al. 2001). Overall, NT2 remains inadequately understood, and further research is needed to characterise this disorder and define precise borders towards circadian predisposition and sleep deprivation (Matsui et al. 2024).

### 2.3 Idiopathic Hypersomnia

IH is likely a heterogeneous disorder, initially classified in two subtypes (with or without long sleep time), characterised by EDS with sleep inertia, with unknown pathophysiology. To date, no specific biological biomarker for IH has been identified. Hypotheses have suggested disruptions in circadian or homeostatic regulation, as well as dysfunction in one or more arousal systems. A potential impairment of the GABAergic signalling pathway was suspected (Rye et al. 2012); however, no active component was detected in the CSF, nor were significant differences observed compared to other CDH. Moreover, these findings were not replicated by another group (Dauvilliers et al. 2016). Another study comparing CSF levels of monoamines, their metabolites, and trace amines between patients with CDH (NT1, NT2, IH and controls) have not identified significant differences (Barateau, Jaussent, et al. 2021). A familial component has often been reported in IH. However, no genetic factor has been identified in IH thus far, even though recent findings support a genetic predisposition and identify pathways involved in the pathogeny of IH (variant in the PER3 gene) (Cherasse et al. 2024). A dysregulation of sleep processes, implicating disruptions in homeostatic or circadian rhythms was reported (Sforza et al. 2000). Melatonin secretion through salivary samples indeed revealed a 2‐h delay in melatonin release in IH, in a small case–control study (Nevsímalová et al. 2000). A reduced expression amplitude of clock genes (BMAL1, PER1, and PER2) was shown in dermal fibroblasts from IH patients, with a prolonged phase of BMAL1 expression (Materna et al. 2018; Lippert et al. 2014). However, a recent study assessing the circadian rhythm of core body temperature in IH patients, non‐specified hypersomnia cases, and healthy controls under a controlled 32‐h bedrest protocol found no differences in circadian phase or amplitude between groups (Adam et al. 2025). This recent study suggests that IH pathophysiology is not primarily driven by circadian dysfunction. Two studies investigating slow‐wave activity (SWA) power in well‐defined IH patients without long sleep duration reported a significant reduction in SWA compared to controls, associated with a decrease in N3 (Sforza et al. 2000; Pizza, Ferri, et al. 2013). However, the natural decline in SWA across sleep cycles remained intact in IH patients. These findings indicate that while homeostatic regulation is preserved, patients with IH may experience lower sleep pressure at sleep onset. This hypothesis needs to be further studied.

### 2.4 Kleine‐Levin Syndrome

The pathophysiology of KLS is also poorly understood. Some studies suggest recurrent primary hypothalamic dysfunction often preceded by acute flu‐like syndrome or upper airway infection and mediated by immune mechanisms (Dauvilliers et al. 2002). Post‐mortem studies are scarce, but signs of encephalitis in thalamic and hypothalamic regions have been detected in few patients (Fenzi et al. 1993; Takrani and Cronin 1976; Koerber et al. 1984; Carpenter et al. 1982; Arnulf et al. 2005). Functional brain imaging during symptomatic episodes consistently shows metabolic alterations, including mainly the thalamus, hypothalamus, mesial temporal, and frontal lobes, but with variable results across studies (Dauvilliers, Bayard, et al. 2014). The young age of onset and the frequent presence of infectious triggers suggest a possible autoimmune process. The most widely accepted hypothesis is that KLS is a transient, recurrent encephalopathy, localised but multifocal (Dauvilliers et al. 2002). Mild reductions in CSF orexin‐A concentrations during symptomatic episodes support this hypothesis (Lopez et al. 2015; Wang et al. 2016). Despite advancements in understanding other sleep disorders, the pathophysiology of KLS remains elusive, and we recently showed an absence of microglial activation in KLS (Barateau, Krache, et al. 2024b). Yet, in a recent study using a proteomic approach, researchers had identified several differentially expressed proteins in CSF and serum, with a predominant dysregulation of microglial axis proteins in CSF (Hédou et al. 2022). However, serum cytokine analyses have not shown consistent differences between symptomatic and asymptomatic phases (Kornum et al. 2015). Another hypothesis posits that KLS may result from a structural or neurodevelopmental brain disorder. A recent genome‐wide association study (GWAS) revealed a significant signal in the TRANK‐1 region, with stronger associations in patients reporting birth‐related complications (Ambati et al. 2021). This study suggests a link between KLS and perinatal complications, similar to findings observed in schizophrenia. Despite ongoing research, KLS remains a poorly characterised and understudied disorder, necessitating further investigations to clarify its pathophysiology and identify reliable diagnostic biomarkers.

## 3 Clinical Features and Diagnosis

### 3.1 Clinical Features

EDS is defined by the inability to stay awake and alert during the major waking periods of the day, resulting in periods of irrepressible need for sleep or unintended lapses into drowsiness or sleep (American Academy of Sleep Medicine 2023). It is the core symptom of CDH and present differently across the disorders. In narcolepsy, especially in NT1, intense daytime sleepiness can already arise in the morning, mostly in monotonous situations but also in more active ones. EDS is poorly resistible with frequent refreshing naps associated with dreaming. Resisting sleepiness may cause automatic behaviours, patients continuing ongoing activities with impaired consciousness and subsequent amnesia (Guilleminault et al. 1975; Barateau et al. 2023). In patients with IH, EDS can manifest with the tendency to fall asleep in situations of variable activation. Typically, in IH, sleepiness is associated with prolonged sleep time (considered abnormal > 11/24 h), which is scarcely restorative (Vernet and Arnulf 2009). Sleep inertia is a key feature of IH, experienced as an extreme awakening difficulty with confusion, disorientation, poor motor coordination, and repeated return to sleep, and manifests at awakening from both nocturnal and daytime sleep making daytime napping unrefreshing (Vernet et al. 2010; Anderson et al. 2007; Dauvilliers, Bogan, et al. 2022).

Cataplexy is the pathognomonic symptom of NT1, consisting of brief, sudden, and transient loss of muscle tone, triggered by emotions, typically positive, with preserved consciousness (Bassetti et al. 2019; Dauvilliers, Siegel, et al. 2014). In paediatric NT1, cataplexy can be persisting fluctuating hypotonia typically involving the face (*facies cataplectica*) without emotional stimulation, possibly associated with superimposed hyperkinetic movements (Plazzi et al. 2011). Since cataplexy may be not directly observed in clinical practice, its recognition mostly relies on reports by the patient or caregivers. According to ICSD‐3‐TR, physicians should classify cataplexy as ‘typical’ or ‘atypical’ based on the features of the reported attacks (distribution, duration, frequency, and triggers), with ‘atypical’ cataplexy considered more probably a possible mimic (American Academy of Sleep Medicine 2023). Standardisation to document cataplexy has been proposed (Vandi et al. 2019), as well suggest a possible automatic detection from video recordings (Bartolini et al. 2018).

In narcolepsy, especially in NT1, disrupted nocturnal sleep (DNS) is a disabling symptom that is gaining more and more attention in clinical practice and research (Barateau, Chenini, et al. 2024). Patients complain of multiple short awakenings and overall poor sleep quality during the night, which is not directly explained by sleep‐disordered breathing, periodic limb movement during sleep, or parasomnias, which can co‐occur in the context of narcolepsy (Maski et al. 2022). DNS, however, should not be a prominent symptom outside of NT1 among central CDH, as it may indicate the presence of another intrinsic sleep disorder underlying the EDS. Sleep paralysis and hypnagogic/hypnopompic hallucinations are considered symptoms of dissociation between elements of REM sleep and wakefulness (Bassetti et al. 2019; Antelmi et al. 2016; Dauvilliers, Billiard, et al. 2003), characteristics of narcolepsy and more frequent in NT1 than in NT2 (Barateau, Chenini, et al. 2024; Dauvilliers et al. 2020), but occurring also in the general population as benign phenomena.

KLS is a recurrent‐remittent hypersomnia disorder with a distinct clinical presentation. During the acute phases (lasting often 8–20 days) patients exhibit a profound increase in total sleep duration (often 16–20 h) from which they are difficult to awaken. Intense sleep inertia, vivid dreaming, and hallucinations are common. During wakefulness, patients can experience eating and sexual disinhibition, cognitive impairments with mental slowness, amnesia, and spatiotemporal disorientation, apathy, or irritability. Altered perceptions of self and environment are frequently described, with derealisation, further pointing to a sub‐wakefulness condition. The episodes resolve spontaneously (Lavault et al. 2015; Arnulf et al. 2008). Outside the acute phase, the patients are mostly asymptomatic, even though subtle cognitive impairment has been observed, and psychiatric disturbances can appear (Uguccioni et al. 2016; Groos et al. 2018). At the onset of the disease, typically during adolescence, frequent episodes can be observed, which then become less frequent over the years and often resolve completely later in life (Arnulf et al. 2008).

### 3.2 Disease Course, Comorbidities, and Assessment

Narcolepsy and IH are chronic disorders frequently arising at a young age with symptoms affecting everyday life. However, the long‐term course of these diseases has not been fully elucidated yet. The age of onset of narcolepsy is bimodal, with the highest incidence from childhood to early adulthood and a second peak in the third decade (Bassetti et al. 2019; Dauvilliers et al. 2001). The phenotype of NT1 can evolve from childhood to adulthood, especially in terms of severity and semeiology of cataplexy and in the expression of EDS (Plazzi et al. 2018; Pizza, Franceschini, et al. 2013; Serra et al. 2008). Diagnostic delay remains crucial, and may influence overall burden calling for improving disease awareness (Ingravallo et al. 2012). Several works disclosed the burden of EDS in CDH at different ages of life, despite in the current review we did not specifically addressed this aspect. NT1 is also associated with a burden of comorbidities: metabolic disorders (i.e., dyslipidemia, obesity, and diabetes), hypertension, increased risk of cardiovascular events (Black et al. 2017; Jennum et al. 2013; Jennum, Pickering, et al. 2017; Ohayon 2013; Barateau and Dauvilliers 2023; Ben‐Joseph et al. 2023; Poli et al. 2009), and psychiatric disorders (mainly depression and anxiety) (Ohayon 2013; Barateau et al. 2020; Dauvilliers et al. 2009; Biscarini, Bassi, et al. 2024). In children with NT1, weight gain and precocious puberty can also occur close to disease onset (Jennum, Pickering, et al. 2017; Plazzi et al. 2006; Poli et al. 2013; Plazzi et al. 2018; Ponziani et al. 2016), and attention‐deficit/hyperactivity disorder can overlap (Lecendreux et al. 2015; Simoncini Malucelli et al. 2024; Inocente et al. 2014; Rocca et al. 2016). Mechanisms of these multiple comorbidities are unclear, as they could depend on hypocretin deficiency, on the prescribed medications, or be consequences of the functional impairment caused by the chronic disease. It is also unclear whether the disease and its comorbidity burden carry an increased mortality risk, as some studies on national registries of diseases suggested (Ohayon et al. 2014; Jennum, Thorstensen, et al. 2017).

IH typically appears from childhood to early adulthood. The natural progression of NT2 and IH is unclear. Some patients diagnosed with NT2 may later develop cataplexy, or with CSF‐HCRT1 deficiency, thus requiring re‐labelling as NT1 (Baumann‐Vogel et al. 2021; Andlauer et al. 2012). Others may show a phenotypic overlap with IH (Bassetti and Aldrich 1997). Thus, the distinction between these two entities has been questioned, and the spectrum of narcoleptic borderland to encompass central hypersomnolence disorders without CSF‐HCRT1 deficiency has been proposed (Lammers et al. 2020; Fronczek et al. 2020). A cluster analysis on a wide European cohort showed that patients without cataplexy were differentiated based on the presence of specific features, such as sleep inertia, refreshing/unrefreshing naps, hypnagogic hallucinations, and sleep paralysis, rather than on the standard diagnostic MSLT criteria of narcolepsy/IH (Gool et al. 2022). Similar results were previously provided by cluster analysis on narcolepsy with and without cataplexy and IH with and without long sleep time (according to the definitions of ICSD 2nd edition) (Šonka et al. 2015). Once established, IH is considered stable in severity and long‐lasting, although spontaneous remission over the years has been observed in up to one‐third of patients (Anderson et al. 2007; Kim et al. 2016). Mood disorders are also associated with NT2 and IH, albeit to a lesser degree than NT1 (Dauvilliers et al. 2009). It is not yet known whether these patients should remain NT2 and IH or rather receive a diagnosis of hypersomnia associated with a psychiatric disorder.

Various scales have been developed for the clinical characterisation of CDH. Some are meant to be disease specific. The Narcolepsy Severity Scale (NSS) and the Ullanlinna Narcolepsy Scale are used to measure the severity of narcolepsy (Dauvilliers et al. 2017, 2020; Sarkanen et al. 2019). A modified version of NSS has been recently validated in the paediatric population (NSS‐P) (Barateau, Lecendreux, et al. 2021). NSS was developed to quantify core NT1 symptoms, but an alternative version (NSS‐2) for NT2 has been recently developed (Barateau, Chenini, et al. 2024). The Idiopathic Hypersomnia Severity Scale was developed to assess the symptoms of IH in adults (Rassu et al. 2022). Other scales are employed to quantify a specific symptom. For measuring chronic EDS, the Epworth Sleepiness Scale (ESS) (Johns 1991) and its version adapted for children and adolescents (ESS‐CHAD) (Grace Wang et al. 2017) are the most used. The Karolinska Sleepiness Scale and the Stanford Sleepiness Scale quantify state sleepiness at assessment time; therefore, they can support self‐reported daytime sleepiness during the polysomnographic procedures but do not address disease severity (Hoddes et al. 1972; Åkerstedt and Gillberg 1990). Tools to quantify psychological distress are also used in CDH, such as the Beck Depression Inventory for mood disorders (Barateau et al. 2020; Beck et al. 1996) and the State–Trait Anxiety Inventory for anxiety (Biscarini, Bassi, et al. 2024; Spielberger et al. 1983; Barateau, Lopez, et al. 2022). Moreover, several non‐sleep related symptoms are referred to as having a significant impact on everyday life in CDH, calling for further development of patients' related outcomes measures to better address overall quality of life (Schokman et al. 2022).

While IH is diagnosed more frequently in females (Acquavella et al. 2020; Arnulf et al. 2023), narcolepsy has a similar prevalence in males and females (Bassetti et al. 2019). However, it is still an open question whether sex or gender influences the different phenotypes (Barateau et al. 2025; Ferrazzini et al. 2025).

### 3.3 Neurophysiological Assessment

Neurophysiological tests are mandatory to diagnose CDH. To apply the current criteria, a nocturnal PSG and an MSLT on the following day represent the core diagnostic protocol in the suspicion of narcolepsy and IH. MSLT is composed of four to five trials, every 2 h starting at 9.00 a.m., in which the patient is invited to fall asleep in a bed in a dark room, with a PSG recording. To be performed properly, stimulating substances and sleep between the trials should be avoided. Each trial should be terminated after 20 min if the patient does not sleep, or 15 min after sleep onset (Krahn et al. 2021; Maski et al. 2024). A mSL ≤ 8 min combined with ≥ 2/5 SOREMPs during MSLT (and including the possible SOREMP occurring during the nocturnal PSG performed the night before the test) is a diagnostic criterion for narcolepsy (both NT1 and NT2), while mSL ≤ 8 min and < 2/5 SOREMPs can diagnose IH (American Academy of Sleep Medicine 2023). In children, alternative MSLT criteria for NT1 were validated; mSL ≤ 8.2 min or ≥ 2 SOREMPs resulted as accurately as the combination of mSL ≤ 8 min and ≥ 2 SOREMPs (Pizza et al. 2019).

Nocturnal PSG is required to exclude other sleep disorders (such as sleep‐disordered breathing or periodic limb movement during sleep) that can explain EDS mimicking NT2 or IH, and to document a proper amount of nocturnal sleep (at least 6 h/night) needed to consider the following MSLT reliable (American Academy of Sleep Medicine 2023; Krahn et al. 2021; Maski et al. 2024). A nocturnal SOREMP does not only enter in the count of the MSLT SOREMP to confirm narcolepsy diagnosis (either NT1 or NT2), but, in the presence of ‘typical’ cataplexy, can be the only neurophysiological marker for NT1 diagnosis, according to the latest ICSD‐3‐TR (American Academy of Sleep Medicine 2023), given its high specificity for CSF‐HCRT1 deficiency (Reiter et al. 2015; Andlauer et al. 2013). When IH is suspected, recording long sleep time is necessary, as MSLT can be negative in up to 60% of cases (Fronczek et al. 2020). In the current criteria, it is now accepted that the documentation of at least 11 h out of 24 h of sleep by means of actigraphy or prolonged PSG recording can document an excessive need of sleep and confirm IH diagnosis (American Academy of Sleep Medicine 2023; American Academy of Sleep Medicine 2014). Albeit this evidence comes from a specific protocol with continuous 24‐h PSG performed after the standard nocturnal PSG + MSLT assessment (Vernet and Arnulf 2009), no recommendations on the continuous recording procedure are given in the criteria (e.g., home vs. in laboratory recording, indication to perform multiple naps at specific time of the day, etc.).

Overall, two main features are innovative in the current criteria, namely the addition of nocturnal PSG SOREMP as a diagnostic feature for NT1 and the determination of 24‐h total sleep time for IH. However, in the last two decades, a series of studies suggest the possibility to implement alternative objective neurophysiological markers to better qualify narcolepsy and IH, extracting information from the MSLT, from continuous PSG, and from nocturnal PSG.

The MSLT can better differentiate the ‘narcoleptic’ profile. Indeed, when considering the SOREMP occurrence, we may also consider the sleep stage sequence leading to REM. While NT1 patients most frequently enter SOREMP directly from wakefulness or non‐REM sleep stage 1 and with a short REM latency (Marti et al. 2009; Drakatos et al. 2013), a marker also potentially correlated with good response to treatment (Drakatos et al. 2016), patients with insufficient sleep syndrome have higher REM latency and most frequently enter SOREMP passing through sleep stage 2 (Marti et al. 2009; Drakatos et al. 2013). Moreover, shorter REM sleep latency at the MSLT can help to differentiate NT1 from NT2 either alone (Zhang et al. 2018) or in combination with the percentage of time spent in REM and with the sleep stage sequence analysis (Murer et al. 2017). A recent study not only confirmed these findings but showed that the absolute time spent in REM sleep during the MSLT can be a marker useful to identify hypocretin deficiency among subjects with hypersomnia and with narcolepsy (Lopez et al. 2023). In contrast, very few data addressed the utility of the MSLT for IH diagnosis. A single study disclosed that a sleep onset profile characterised by fluctuations between sleep (defined as a single epoch of sleep stage 1) and wakefulness before the onset of a sustained sleep condition (three consecutive sleep stage 1 epochs of an epoch of any other sleep stage) can positively identify IH (Pizza et al. 2011). Conversely, several data showed the poor test–retest reliability of standard MSLT data for CDH diagnosis in non‐cataplectic/hypocretin deficient patients (Lopez et al. 2017; Trotti, Staab, et al. 2013; Um et al. 2017; Ruoff et al. 2018). These results call for considering the implementation of additional neurophysiological markers to improve diagnosis and differential diagnosis and the need to validate their reliability also in the paediatric population.

In parallel, mounting data highlights the possibility of identifying NT1 by using nocturnal PSG alone. Aside from nocturnal SOREMP (American Academy of Sleep Medicine 2014, 2023), the specificity of sleep stage sequencing leading to SOREMP directly from sleep stage 1 or wakefulness in NT1 has been confirmed in adults (Drakatos et al. 2013; Liu et al. 2015). Nocturnal sleep of NT1 patients also showed an instability that can be easily quantified by indexing the overnight transitions between wakefulness and sleep or between non‐REM and REM sleep (Sorensen et al. 2013), a marker that, coupled with the percentage of time spent in non‐REM sleep stage 1 and occurrence of SOREMP, can be used to diagnose NT1 in adults (Pizza et al. 2015). In this context, NT1 not only showed an increased nocturnal sleep instability but also an increase in the likelihood of shifting from REM sleep to non‐REM sleep useful for diagnostic purposes (Ferri et al. 2016). In children with NT1, the combination of nocturnal SOREMP with high sleep instability (quantified as the index of transitions from sleep to wakefulness or sleep stage 1) (Maski et al. 2020), or with an objective quantification of muscle tone during REM sleep, was confirmed as possible diagnostic markers of NT1 (Silvani et al. 2021). Automated quantitative PSG analyses disclosed the possibility to characterise sleep in terms of hypnodensity (i.e., a metric describing the probability of occurrence of each sleep state for each epoch) have been performed. These studies showed promising results for distinguishing CDH, based on the concept that, in humans with NT1, sleep stage boundaries are less defined (Stephansen et al. 2018; Olsen et al. 2017; Cesari et al. 2023; Vilela et al. 2024). These findings mirror the evidence from animal models (Diniz Behn et al. 2010) and suggest the need to implement automatic sleep stage scoring as a possible perspective in NT1 diagnosis, as well as the need to validate the results in the paediatric population.

Nocturnal sleep, instead, has not been employed alone to identify non‐orexin deficient CDHs but mostly to distinguish NT1 sleep from other CDHs. In NT2 the nocturnal SOREMP is rarer than in NT1 and sleep is reported to be less fragmented. In IH, SOREMPs should be absent and sleep structure is typically reported as normal, with long sleep time, high efficiency, and more representation of slow wave sleep at the end of the night. A meta‐analysis of 10 studies comparing PSG in IH and healthy controls showed increased total sleep time and REM sleep percentage, and reduced sleep latency and non‐REM stage 3 (Plante 2018). More detailed analysis of sleep dynamics in IH suggests some peculiar features. In adolescents with IH, non‐REM stage 2 appeared more stable and non‐REM stage 3 bouts were shorter than sleepy controls (Maski, Colclasure, et al. 2021). In IH, a less fragmented sleep (less awakenings, lower non‐REM stage 1 percentage, less stage shifts, and higher REM stage percentage) was associated with reported unrefreshing naps (Mombelli et al. 2023). A deeper description of the nocturnal sleep in IH and NT2 would benefit the understanding of these disorders.

Daytime and combined daytime and nighttime PSG recordings are protocols that are raising interest in the field and their role could be expanded. Primarily, prolonged daytime‐nighttime PSG is needed to record long sleep time in IH (Evangelista et al. 2024). Different protocols are available, of variable duration and with variable instructions. In the international guidelines, no detailed indications are provided (American Academy of Sleep Medicine 2023) and no comparative studies have been produced. The protocol published by the Sleep Center in Paris‐France establishes a continuous PSG recording with an ad libitum night sleep, followed by two opportunities for uninterrupted naps. With this protocol, 660 min (11 h) was identified as the optimal threshold to define long sleep time in IH (Vernet and Arnulf 2009). At the Sleep Center of Montpellier‐France, patients are recorded for a night PSG, a modified MSLT, and then for 32 h of continuous PSG in bedrest conditions and without external circadian synchronisers to reach the maximum spontaneous amount of sleep. With this procedure, the authors validated a cutoff of 19 h as the best to diagnose IH in adults (Evangelista et al. 2018). At the Narcolepsy Center of Bologna‐Italy, the procedure, applied in a hospital setting to all suspicions of CDHs, comprises ad libitum sleep opportunity for 48 h of continuous PSG recording with a portable device (Pizza, Moghadam, et al. 2013). No specific threshold for long sleep time is provided to date with this protocol, but the recording of daytime SOREMPs during spontaneous naps proved to identify NT1 in adults and children with high accuracy (Pizza, Moghadam, et al. 2013; Pizza et al. 2024), and was recently demonstrated to be superior to nocturnal SOREMP (Biscarini et al. 2025). These findings pave the way to the possibility of testing prolonged PSG recordings in a home setting to improve the diagnostic process of these rare disorders.

The use of wearable devices will likely expand in the next few years as tools for the diagnosis and characterisation of CDHs, as in other branches of sleep medicine (Liguori et al. 2023). Actigraphy is currently recommended in clinical practice to rule out potential confounding factors of CDH, such as circadian rhythm disorders or chronic sleep deprivation, and can be employed to estimate sleep duration > 11 h for the diagnosis of IH (American Academy of Sleep Medicine 2023). To strengthen its use, studies on adult and paediatric NT1 demonstrated that actigraphy can record reduced daytime activities, naps, and increased nocturnal motor activity reflecting the sleep fragmentation (Filardi et al. 2016; Alakuijala et al. 2016; Leger et al. 2018). These metrics showed the potential utility of actigraphy to distinguish different NT1 from controls with and without hypersomnolence (Filardi et al. 2015; Torstensen et al. 2021), even though the issue of conventional actigraphic algorithms overestimating total sleep time in IH and underestimating nighttime sleep in narcolepsy should be addressed (Biscarini, Vandi, et al. 2024). Wearables with multi‐sensor activity trackers that integrate accelerometry and heart rate are also gaining interest in the field, as they can estimate sleep fragmentation and shorter sleep latency typical of NT1 in long‐term recordings (Gnarra et al. 2024), despite a study showing a failure in detecting SOREMPs (Cook et al. 2019). These tools and related measures would be potentially useful in the context of KLS to quantify total sleep time during the acute phase compared to asymptomatic periods.

Besides diagnostic purposes, neurophysiology can be used to objectively quantify the symptoms of CDHs, in order to define phenotypes, stratifying severity and potentially measuring response to treatment. The maintenance of wakefulness test (MWT) is employed to record the ability to resist sleepiness in a monotonous setting, and it is widely used in clinical practice and in pharmacological trials to quantify improvement of EDS and, in some countries, to assess EDS in contexts where it can represent a safety issue (e.g., for driving license) (Krahn et al. 2021; Arand et al. 2005). In the context of CDHs, MWT shows reduced sleep latency that improves after treatment (Bijlenga et al. 2022). However, no formally accepted normative values exist for MWT (per session and averaged per test) and data on test results in CDH basal conditions have rarely been reported. MWT could provide additional EEG‐derived biomarkers of sleep pressure, such as microsleep, hypnodensity, or spectral analysis to quantify more precisely EDS in CDHs (Tracey et al. 2024). Other features of CDH do not have established objective markers. A PSG‐based definition of DNS is lacking, although the complaint of DNS has been associated with sleep fragmentation markers in NT1 (e.g., sleep–wake transitions, more wake and sleep bouts, and longer wake after sleep onset) (Barateau, Lopez, et al. 2022; Maski et al. 2020). The improvement of nocturnal sleep and reduced daytime sleep with sodium oxybate was shown with actigraphy in children with NT1 (Filardi et al. 2018). In IH, sleep inertia has been studied with the psychomotor vigilance test (PVT) as a reliable objective marker in pharmacological trials (Evangelista et al. 2022; Trotti et al. 2015, 2022). Finally, several approaches were used to document and describe cataplexy with a video polysomnography during emotional stimulation, disclosing the reliability of facial video recording during laughter to identify cataplexy from other mimics (Pizza et al. 2018). Based on these premises, we suggest the utility of developing a video automatic detection approach for cataplexy that may support differential diagnosis with attack mimics.

### 3.4 Cerebrospinal Fluid Orexin/Hypocretin Assessment

The current diagnostic criteria for NT1 and NT2, as outlined in the ICSD‐3‐TR (American Academy of Sleep Medicine 2023), are based on clinical assessment, neurophysiological testing, and CSF orexin‐A level measurements. Thresholds for orexin deficiency were established in 2002 and defined as below 110 pg/mL, normal above 200 pg/mL, and intermediate between 110 and 200 pg/mL (Mignot et al. 2002). Despite significant advancements in understanding narcolepsy, these cut‐offs have seldom been reassessed (Van Der Hoeven et al. 2022), even as classification criteria have evolved over time (American Academy of Sleep Medicine 2005, 2014, 2023). Interestingly, individuals with intermediate orexin levels are rare and have been the subject of limited research. In a recent study, clinical and neurophysiological profiles of such patients were characterised by analysing data from two specialised centres (Postiglione et al. 2022). Future research should aim to re‐evaluate the established orexin deficiency thresholds via alternative assessment methods (e.g., mass spectrometry (Hirtz et al. 2016)) and further explore the relationship between orexin levels and clinical as well as neurophysiological features, particularly in individuals with intermediate CSF orexin levels.

## 4 Management

Available therapies for CDH are symptomatic and include nonpharmacological and pharmacological approaches. No curative therapy exists for either narcolepsy, IH, or KLS. In 2021, the European Academy of Neurology, European Sleep Research Society, and European Narcolepsy Network published the most recent European guidelines for the treatment of narcolepsy in adults, with a recent update in children (Bassetti et al. 2021). In the same year, the American Academy of Sleep Medicine (AASM) published guidelines for the treatment of CDH in adults and children, sharing many similarities (Maski, Trotti, et al. 2021).

### 4.1 Current Treatment for Narcolepsy

Guidelines support the use of pharmacological symptomatic treatment for long‐term control of narcolepsy, in both adults and children. The choice of first‐line medication is driven by the clinical picture and the presence of core symptoms (Bassetti et al. 2021; Maski, Trotti, et al. 2021).

Psychostimulants are effective in improving EDS in both NT1 and NT2 by enhancing dopaminergic transmission (i.e., modafinil and armodafinil) or noradrenergic and dopaminergic activity (i.e., solriamfetol, methylphenidate, and amphetamine derivates). Modafinil/armodafinil and solriamfetol are among the first‐line options in adults, with strong recommendations in both European and American guidelines (Bassetti et al. 2021; Maski, Trotti, et al. 2021). Pitolisant is also a first‐line option. This drug is an inverse agonist of the presynaptic histamine H3‐receptor that mainly increases CNS histaminergic transmission, effective in improving EDS, cataplexy, and hallucinations in patients with NT1, both adults (Dauvilliers, Roth, et al. 2023; Roth et al. 2022; Bogan et al. 2021) and children (Dauvilliers, Lecendreux, et al. 2023). Amphetamine derivatives and methylphenidate are second‐line choices for EDS in adult narcolepsy, with conditional recommendations due to potential major side effects and lack of evidence with randomised controlled trials (Bassetti et al. 2021; Maski, Trotti, et al. 2021). Sodium oxybate is a gamma aminobutyric acid (GABA) B‐receptor agonist that, taken twice at night, promotes slow‐wave sleep and probably modulates noradrenergic and dopaminergic neurons during the daytime. Sodium oxybate promotes depth and continuity of nocturnal sleep, improving cataplexy and EDS. Thus, it is accounted among first‐line therapies for EDS, cataplexy, and DNS in adult and paediatric NT1 (Bassetti et al. 2021; Maski, Trotti, et al. 2021). Recently, alternative formulations of oxybate with a lower content of sodium (lower‐sodium oxybate, LXB) and with longer action that can be administered once‐nightly (once‐nightly sodium oxybate, ON‐SXB) have been demonstrated to be effective in controlling symptoms of narcolepsy in adults (Dauvilliers, Roth, et al. 2023; Roth et al. 2022; Bogan et al. 2021; Dauvilliers, Šonka, et al. 2022). Antidepressants (especially serotonin‐noradrenalin re‐uptake inhibitors, serotonin re‐uptake inhibitors, and tricyclics) are effective in controlling cataplexy and have a weak recommendation for combination with a wake‐promoting agent in adult and paediatric NT1 in European Guidelines (Bassetti et al. 2021), but caution should be used for the possible occurrence of cataplexy rebound up to cataplectic status at withdrawal (Poryazova et al. 2005).

Under 6‐year‐old, no pharmacological treatments are approved for narcolepsy by the main regulatory agencies. The Food and Drug Administration (FDA) and the European Medicines Agency (EMA) approved the use of pitolisant for patients with NT1 and NT2 older than 6 years, and sodium oxybate for patients with NT1 older than 7 years. The FDA additionally approved ON‐SXB for adult narcolepsy and LXB for adult and paediatric NT1 (without a specific clinical trial in children). For the treatment of EDS in adults with narcolepsy, modafinil, solriamfetol, pitolisant, and methylphenidate are approved by the EMA and FDA, and armodafinil and amphetamine derivatives are approved by the FDA only. Antidepressants are considered off‐label for the treatment of cataplexy in all ages. Available pharmacological treatments for narcolepsy are summarised in Table 1.

**TABLE 1:** Pharmacological treatments for narcolepsy.

Drug	Neurobiological mechanism	Targeted symptoms	Quality of evidence and strength of recommendation	Approval
Modafinil	Dopamine reuptake inhibition	EDS	Moderate in adults, very low in children Strong for adults (AASM, EU) Weak for children (AASM, EU)	EMA, FDA Approved for narcolepsy in adults
Armodafinil	Dopamine reuptake inhibition	EDS	Moderate in adults, very low in children Weak for adults (AASM)	FDA Approved for narcolepsy in adults
Methylphenidate	Dopamine and norepinephrine reuptake inhibition	EDS	Low in adults, very low in children Weak for adults (AASM, EU) Weak for children (EU)	EMA, FDA Approved for narcolepsy in adults
Sodium oxybate	GABA‐B receptor agonist	EDS, cataplexy, DNS	Moderate in adults, low in children Strong for adults (AASM, EU) Strong for children (EU) Weak for children (AASM)	EMA, FDA Approved for narcolepsy with cataplexy > 7 yo
Low‐sodium oxybate	GABA‐B receptor agonist	EDS, cataplexy, DNS	No recommendations	FDA Approved for narcolepsy with cataplexy > 7 yo
Once‐nightly sodium oxybate	GABA‐B receptor agonist	EDS, cataplexy, DNS	No recommendations	FDA Approved for narcolepsy with cataplexy in adults
Pitolisant	Histamine H3 receptor antagonist/inverse agonist	EDS, cataplexy	Moderate in adults, low in children. Strong for in adults (AASM, EU), moderate for children (EU).	EMA, FDA Approved for narcolepsy > 6 yo
Solriamfetol	Dopamine and norepinephrine reuptake inhibitor	EDS	Moderate in adults, none in children Strong for in adults (AASM, EU)	EMA, FDA Approved for narcolepsy in adults
d‐Amphetamines	Dopamine and norepinephrine reuptake inhibitor	EDS	Low in adults, none in children Weak for adults (AASM, EU), weak for children (EU)	FDA Approved for narcolepsy in adults
Antidepressants (e.g., SSRIs, SNRIs, tricyclics)	Modulation of serotonin/norepinephrine activity	Cataplexy	Low in adults, very low in children Strong for adults, weak for children (EU)	Off‐label
Orexin‐receptor‐2 agonists	Orexin‐receptor 2 agonist	EDS, cataplexy	No recommendation yet	Not available
Immune based‐therapy (immunoglobulin, plasmapheresis, steroids, monoclonal antibodies)	Acting on the supposed autoimmune process	Suppose to modify disease course	Very low in adults and children Strong against in adults and children (EU)	Not approved and even not recommended

*Note*: Quality of evidence is presented adapted from 2021 American Academy of Sleep Medicine (AASM) and 2021 European Academy of Neurology/European Sleep Research Society/European Narcolepsy Network (EU) Guidelines (Bassetti et al. 2021; Maski, Trotti, et al. 2021). Strength of recommendations is adapted from 2021 AASM and/or 2021 EU Guidelines. Current approval state by regulatory drug agencies (European Medicines Agency—EMA, and Food and Drug Administration—FDA) is presented.

Abbreviations: AASM = American Academy of Sleep Medicine, ADHD = attention deficit hyperactivity disorder, CNS = central nervous system, EDS = excessive daytime sleepiness, EMA = European Medicines Agency, FDA = Food and Drug Administration, GABA = gamma aminobutyric acid, RCT = randomised controlled trial, SNRI = serotonin‐norepinephrine reuptake inhibitor, SSRI = selective serotonin reuptake inhibitor.

### 4.2 Current Treatment for IH

High‐quality evidence on pharmacological therapy for IH is scarce. Modafinil improved EDS in a few small randomised control trials (RCT) in patients with IH older than 16 years (Inoue et al. 2021; Philip et al. 2014) and in retrospective studies (Anderson et al. 2007; Ali et al. 2009). In the AASM guidelines, it is recommended to treat adults with IH (Maski, Trotti, et al. 2021). LXB was proven to improve EDS, sleep inertia, and overall disease severity in an RCT on 154 adult patients with IH, regardless of the presence of long sleep time, once or twice nightly administration, or other ongoing treatments for IH (Dauvilliers, Arnulf, et al. 2022). LXB was not included in the AASM Guidelines, which were published before the RCT. In retrospective observational studies, other medications have been reported to improve EDS in IH: sodium oxybate (Pascoe et al. 2019; Leu‐Semenescu et al. 2016), methylphenidate (Ali et al. 2009), clarithromycin (Trotti, Saini, et al. 2013), flumazenil (Trotti et al. 2016), and pitolisant (Leu‐Semenescu et al. 2014). Clarithromycin (antibiotic and negative modulator of GABA‐A‐receptor) was also tested in a small RCT and improved subjective EDS without reaching the primary endpoint of improving reaction time on PVT (Trotti et al. 2015). In a recent double‐blind, parallel‐group, placebo‐controlled trial including 45 patients with a clear diagnosis of IH, sodium oxybate resulted in a clinically meaningful improvement, reducing excessive sleepiness on ESS, improving wakefulness on MWT, and decreasing IH severity on IHSS after 8 weeks (Dauvilliers, Chenini, et al. 2025). Sodium oxybate, methylphenidate, clarithromycin, and pitolisant are suggested as second‐line treatments for adult IH by the AASM guidelines, while flumazenil is not recommended (Maski, Trotti, et al. 2021). LXB is the only medication approved by the FDA for treating EDS in adult IH, while no drugs are currently approved in Europe for IH.

### 4.3 Current Treatment for KLS

For KLS, being extremely rare, no RCT has been published, and no labelled treatments are available. During the episodes, symptomatic therapy can be prescribed to control neuropsychiatric manifestations. An open‐label study showed a reduction in the duration of long‐lasting episodes (defined as episodes lasting more than 7 days) in patients treated with intravenous methylprednisolone during the episode (Léotard et al. 2018). In another open‐label study, chronic preventive treatment with lithium has shown a reduction in the frequency and length of episodes (Leu‐Semenescu et al. 2015). In the AASM guidelines, a conditional recommendation was given in favour of lithium in KLS, while evidence on methylprednisolone was deemed insufficient to provide a recommendation (Maski, Trotti, et al. 2021).

### 4.4 Non‐Pharmacological Treatments in CDH

Non‐pharmacological approaches to symptom control should always be considered first and applied alongside symptomatic drugs. In some situations where medication is considered inappropriate, such as pregnancy, non‐pharmacological management approaches are mandatory (Bassetti et al. 2021). In narcolepsy, a key element of treatment is scheduled napping, as it is recommended in European guidelines at any age (Bassetti et al. 2021). Naps should be brief (i.e., 15–20 min) to optimise the refreshing effect of sleep and reduce sleep inertia. Sleep hygiene, regular sleep–wake rhythms, and maintaining regular physical activity are additional recommended practices. Increasing the patients' knowledge of their disease and its mechanisms is mandatory, and adequate information should be provided also to familiar environment. Joining a patients' group can help with coping with the disease burden and its psychological consequences (Bassetti et al. 2021). Psychotherapy such as cognitive‐behavioural therapy (CBT) can benefit the psychological and psychiatric comorbidity of the disease (Franceschini et al. 2021), and ad hoc interventions may improve overall disease burden, also focusing on non‐sleep‐related symptoms (Varallo et al. 2025).

In IH, evidence about nonpharmacological management is less strong than in narcolepsy. Napping can be beneficial in the rare cases that find it refreshing; otherwise, it should be avoided. A study conducted during the COVID‐19 pandemic‐related lockdowns suggested that teleworking, with the consecutive freer schedule of daytime and nighttime sleep, was beneficial to patients with IH (Barateau, Chenini, et al. 2022). Allowing patients to start later at work (e.g., with teleworking) could reduce the impact of sleep inertia.

Rigorous trials on the effect of nonpharmacological therapies in CDH are needed (Varallo et al. 2025). Disease‐specific protocols of CBT and mindfulness‐based interventions have been developed, but their usefulness has not been demonstrated yet (Mundt et al. 2024; Ong et al. 2020).

### 4.5 Perspectives for Management

Given the distinct pathophysiology of NT1, orexin replacement therapy has always represented a perfect treatment option. Among potential candidates, orexin receptor‐2 agonists have shown particular promise in both orexin/ataxin‐3 narcolepsy mouse models and human patients with NT1 and NT2 (Evans et al. 2022). A recent breakthrough in the sleep field demonstrated near‐complete normalisation of sleepiness and cataplexy over an 8‐week period in NT1 patients, though the treatment was associated with some hepatotoxic effects (Dauvilliers, Mignot, et al. 2023). A recent phase 2 trial involving participants with NT1 showed that oveporexton, another oral orexin receptor 2‐selective agonist, significantly improved measures of wakefulness, sleepiness, and cataplexy over a period of 8 weeks. The most common adverse events associated with oveporexton were insomnia, urinary urgency, and urinary frequency, without any cases of hepatotoxicity (Dauvilliers, Plazzi, et al. 2025). Another therapeutic strategy involves preventing the loss of orexin neurons by administering immunomodulatory agents at the onset of NT1, with the aim of altering disease progression. However, attempts to implement such treatments in small, uncontrolled case studies have often led to disappointing outcomes (Barateau et al. 2017), and those therapies are not recommended by experts nowadays (Bassetti et al. 2021; Maski, Trotti, et al. 2021).

## 5 Conclusion

A significant breakthrough in sleep medicine was the discovery that NT1 results from the loss of hypothalamic neurons producing orexin, a neurotransmitter that regulates wakefulness and sleep and can be measured in the CSF currently by RIA. Two decades later, another major advancement has emerged with the development of oral orexin receptor‐2 agonists, offering promising new treatment options for narcolepsy. NT1 has become a valuable model for studying hypersomnolence and its underlying biological mechanisms, providing a foundation for innovations in diagnosis, treatment, and disease understanding. However, it is now crucial to extend this progress to other CDH, particularly the less‐studied NT2, IH, and KLS, whose pathophysiology remains poorly understood. Future efforts should aim not only at advancing pharmacological treatments but also at improving diagnostic precision through the development of objective biomarkers, neurophysiological tools, and wearable technologies. In particular, refining diagnostic criteria, expanding access to effective therapies, and deepening our understanding of disease mechanisms will be essential to optimise patient care. Moving forward, collaborative research efforts and precision medicine approaches hold the potential to improve disease stratification, treatment selection, and overall clinical outcomes in individuals affected by CDH. Finally, non‐sleep related symptoms (not reviewed in the current work) and patients' related outcome measures should be specifically developed to reduce CDH related burden at different ages of life.

## Author Contributions

**Francesco Biscarini:** conceptualization, writing – original draft. **Lucie Barateau:** conceptualization, writing – original draft. **Fabio Pizza:** conceptualization, writing – review and editing. **Giuseppe Plazzi:** conceptualization, writing – review and editing. **Yves Dauvilliers:** conceptualization, validation, supervision, project administration, writing – review and editing.

## Conflicts of Interest

The authors declare no conflicts of interest.

## Data Availability

Data sharing not applicable to this article as no datasets were generated or analysed during the current study.
